# Exposure to Temperature and Insecticides Modulates the Expression of Small Noncoding RNA-Associated Transcripts in the Colorado Potato Beetle, *Leptinotarsa decemlineata* (Coleoptera: Chrysomelidae)

**DOI:** 10.1093/jisesa/ieac004

**Published:** 2022-02-16

**Authors:** Mariem Ben Youssef, Brigitte Christelle Ouédraogo, Pierre Bastarache, Pascal Dumas, Chandra E Moffat, Jessica L Vickruck, Pier Jr Morin

**Affiliations:** 1 Department of Chemistry and Biochemistry, Université de Moncton, 18 Antonine-Maillet Avenue, Moncton, New Brunswick, E1A 3E9, Canada; 2 Fredericton Research and Development Centre, Agriculture and Agri-Food Canada, 850 Lincoln Road, Fredericton, New Brunswick, E3B 4Z7, Canada

**Keywords:** Colorado potato beetle, cold hardiness, heat shock, chlorantraniliprole, clothianidin

## Abstract

The Colorado potato beetle (*Leptinotarsa decemlineata* (Say)) is an insect that can adapt to various challenges, including temperature fluctuations or select insecticide treatments. This pest is also an ongoing threat to the potato industry. Small noncoding RNAs such as miRNAs, which can control posttranscriptionally the expression of various genes, and piRNAs, which can notably impact mRNA turnover, are modulated in insects under different conditions. Unfortunately, information regarding the expression status of key players involved in their synthesis and function is for the most part lacking. The current study thus aims at assessing the levels of such targets in *L. decemlineata* exposed to hot and cold temperatures as well as treated to the insecticides chlorantraniliprole, clothianidin, imidacloprid, and spinosad. Transcript expression levels of Ago1, Ago2, Ago3, Dcr2a, Dcr2b, Expo-5, Siwi-1, and Siwi-2, components of pathways associated with small noncoding RNA production or function, were measured by qRT-PCR and revealed modulation of select transcripts in response to temperature challenges and to select insecticides. RNAi-mediated reduction of Ago2 transcript levels in *L. decemlineata* injected with Ago2-targeting dsRNA and exposed to cold and warm temperatures was also conducted. Changes in survival rates were observed for the latter condition in dsRNA- versus saline-injected insects. These results showcase the differential expression of select targets involved in small noncoding RNA homeostasis and provide leads for the subsequent assessment of their involvement during stress response in *L. decemlineata* using RNAi-based approaches.

The Colorado potato beetle, *Leptinotarsa decemlineata* (Say), is an invasive insect pest that can impact the potato industry worldwide (Weber 2003). Different strategies have been used to reduce its effect on potato crops including various insecticides and biological control. Nevertheless, numerous challenges remain with controlling the damages caused by this insect (Alyokhin et al. 2015, Scott et al. 2015). The beetle demonstrates a marked ability to tolerate various stressors, including chemical compounds or low and high temperature challenges. Characterization of the molecular bases associated with adaptation to these challenges is ongoing. For example, challenges associated with tolerance to freezing in insects, such as the occurrence of mechanical damage, as well as an overview of potential molecular changes, including the modulation of antioxidant enzymes or heat shock proteins, leveraged during such adaptation have been described (Storey and Storey 2012, Toxopeus and Sinclair 2018). Select studies have also notably reported varying expression levels of specific transcripts including ones coding for heat shock proteins in *L. decemlineta* exposed to hot or cold temperatures (Lyytinen et al. 2012, Chen et al. 2016, Dumas et al. 2019). Much information is also available regarding the molecular leads associated with response to insecticides in *L. decemlineata*. Modulation of targets involved in xenobiotic metabolism has been reported in populations that were resistant or sensitive to imidacloprid or chlorothalonil (Clements et al. 2021). Differential expression of transcripts coding for cytochrome P450 (CYP450) genes in a similar context and RNAi-based approaches to evaluate the impact of varying expression of CYP450 targets on insecticide resistance in *L. decemlineata* have also been investigated (Clements et al. 2017, Kalsi and Palli 2017, Kaplanoglu et al. 2017). While these studies explored the molecular responses observed in *L. decemlineata* exposed to various challenges, expression status of transcripts coding for proteins involved in noncoding RNA synthesis has not thoroughly been measured in this insect pest when submitted to temperature changes or insecticides.

Small noncoding RNAs, including microRNAs (miRNAs) and PIWI-interacting RNAs (piRNAs), have been studied to various extent as underlying regulators of stress response in insects. Results showing that adaptation to changes in temperatures can be influenced by temperature-responsive miRNAs and that temperature shifts are associated with an important piRNA response (Fast et al. 2017) are only a few examples of the growing body of literature showcasing small RNA modulation following stress exposure. miRNAs are short, 21–24 nucleotides in length, noncoding RNA molecules that regulate gene expression in a posttranscriptional manner by interacting with mRNA target transcripts. miRNAs are predicted to regulate the expression levels of more than 60% of all protein-coding genes (Friedman et al. 2009). This supports their likely role in regulating adaptation to a variety of conditions including cold temperatures. Accordingly, modulation of a signature of 36 miRNAs in the overwintering rice water weevil, *Lissorhoptrus oryzophilus* (Coleoptera: Erirhinidae) (Yang et al. 2017), as well as the potential role of miR-31-5p during cold acclimation in the wood-boring beetle *Monochamus alternatus* (Coleoptera: Cerambycidae) (Zhang et al. 2020), are select examples of work that highlighted the potential importance of miRNAs in insect cold response. In addition, it is relevant to add that several studies have shown the importance of miRNAs in insects exposed to various insecticides. Modulation of 77 miRNAs in the aphid *Sitobion miscanthi* (Hemiptera: Aphididae) in response to imidacloprid treatment (Zhang et al. 2021) and report of a miRNA that underlies deltamethrin resistance in the mosquito *Culex pipiens pallens* (L.) (Diptera: Culicidae) (Sun et al. 2019) are few examples supporting the link between miRNAs and insecticides response in insects. The cascade associated with miRNA biogenesis has been described substantially (Lucas and Raikhel 2013) and notably involves Exportin-5 (Expo-5), which participates in the export of precursor miRNAs out of the nucleus (Bohnsack et al. 2004), and Argonaute-1 (Ago1), a core component of the RNA-induced silencing complex (RISC) essential for miRNA-mediated regulation of mRNA targets (Okamura et al. 2004). In addition to miRNAs, other short noncoding RNAs, such as piRNAs, have garnered attention in recent years for their underlying roles in regulating a variety of processes. PiRNAs are short, typically 24–30 nucleotides in length, and can preserve germline maintenance in germinal cells. Recently, piRNAs have been associated with diverse functions in somatic cells including epigenetic programming and mRNA turnover (Ku and Lin 2014). The pathway leading to piRNA synthesis notably involves Piwi proteins including Piwi/Siwi and Argonaute-3 (Ago3) that are essential for piRNA maturation and function (Czech et al. 2018). It is interesting to note that the activity of piRNA biogenesis is reduced following changes in temperatures in *Caenorhabditis elegans* (Rhabditida: Rhabditidae) (Belicard et al. 2018). Overall, while multiple reports have presented varying small noncoding RNA levels in insects exposed to diverse conditions, data is limited regarding the expression levels or activity status of molecular players involved in pathways associated with small noncoding RNA synthesis or function. The current work thus tested the hypothesis that reduced or elevated temperatures, as well as insecticides, would be associated with differential expression of transcripts coding for proteins involved in small noncoding RNA expression and function in *L. decemlineata*.

In order to validate this hypothesis, this study was undertaken to quantify transcripts relevant to small noncoding RNA homeostasis in *L. decemlineata* submitted to temperature challenges as well as treated with different chemical compounds of interest for its control. RNAi-based down-regulation of a select target, combined with the effect of this variation on cold and heat response, was also explored. In general, this work sheds the light on the potential impact of select small noncoding RNA-associated targets in response to various conditions in this potato pest.

## Materials and Methods

### Insects

Adult Colorado potato beetles *Leptinotarsa decemlineata* (Say) used for cold, heat, and insecticide exposure were obtained from the Fredericton Research and Development Centre (FRDC) (45° 55′17.5ʺ N 66° 36′01.8ʺ W, Fredericton, New Brunswick, Canada). Insects were all initially collected from potato fields at FRDC that had not been previously exposed to insecticides. Insects were transported to Moncton (New Brunswick, Canada) in plastic containers with potato leaves (var. Kennebec) for insect acclimation for at least 5 d in an incubator (Thermo Fisher Scientific, Waltham, MA) under a 16L:8D photoperiod.

An overview of the overarching study design and research objectives is provided in Supp Fig. 1 (online only). Time-courses for temperature treatments were conducted as follow. Cold exposure was undertaken as described before (Morin et al. 2017a). Temperatures were progressively reduced to levels that were slightly above previously reported low temperature exotherm of −8.8°C observed in active beetles (Boiteau and Coleman 1996). Briefly, adult insects were maintained in an incubator at 15°C for one week. A group of insects, used as controls, was deposited in liquid nitrogen. Temperature was subsequently lowered to 5°C for 2 h after which a group of insects was sampled in liquid nitrogen. Temperature was further lowered to −5°C for 2 h, at which point a group of insects was sampled. Temperature was ultimately increased to 15°C for 24 h to allow for insect recovery followed by sampling. In total, 15 insects were sampled at each temperature. Heat treatment was performed as described before (Dumas et al. 2019). Temperatures were increased to below the 50–55°C range, temperature levels that displayed increasing evidence of insect mortality in adult beetles (Pelletier 1998). Insects were first acclimated to a temperature of 25°C. Control insects were sampled in liquid nitrogen before temperature treatment. Remaining insects were exposed to temperatures of 30°C for 2 h, 35°C for 2 h, and a final temperature of 40°C for 2 h. All insects, 15 per temperature, were sampled in liquid nitrogen following treatments.

Insecticides studied were selected from classes of compounds of interest for *L. decemlineata* management including the neonicotinoids clothianidin and imidacloprid, the anthranilic diamide chlorantraniliprole, and the spinosyn spinosad. Time-courses for treatments with the four insecticides were performed as follow. Adult insects were initially acclimated in an incubator at 25°C. Chlorantraniliprole treatment was carried out as described before (Dumas et al. 2019). A volume of 1 μl (1 µg) of a chlorantraniliprole solution (Sigma-Aldrich, MO), prepared in acetone, was applied topically on the abdomen of adult insects. Insects used as controls were treated with a similar volume of acetone. After 24 h in the incubator, the insects were sampled in liquid nitrogen. Clothianidin treatment was performed by topically applying a volume of 1.25 μl (0.25 µg) of clothianidin (Sigma-Aldrich), dissolved in acetone, to the abdomen of insects. Control insects were treated with the same volume of acetone. Insects were placed back in the incubator for 4 h before sampling as above. The beetles were exposed to imidacloprid according to Morin et al. 2017b. A topical application of 5 μl (0.5 µg) of a solution of imidacloprid dissolved in acetonitrile (Sigma-Aldrich) was carried out on the abdomen of adult insects. A group of insects serving as controls was treated with a comparable volume of acetonitrile. Acetonitrile was used for controlling insects in the imidacloprid exposure time-course specifically as it was the solvent in which this compound was provided by the manufacturer. The insects were then incubated for 24 h at 25°C and then, frozen in liquid nitrogen. Spinosad treatment in adult insects was performed following a protocol described elsewhere (Bastarache et al. 2020). A topical application of 0.5 µl (0.5 µg) of a spinosad solution (Sigma-Aldrich), prepared in acetone, was performed on the abdomen of insects. Control insects were exposed to an identical volume of acetone. The insects, exposed to spinosad or acetone, were subsequently incubated for 4 h and sampled as above. Overall, a total of 15 control and 15 insecticides-treated insects were sampled for time-courses associated with clothianidin, imidacloprid, and spinosad responses while 25 control and 25 treated insects were collected for experiments conducted with chlorantraniliprole. In addition, dosage was determined as previously presented (Morin et al. 2017b, Dumas et al. 2019, Bastarache et al. 2020). Briefly, insecticide exposure was conducted by treating groups of five to eight insects with progressively higher doses of the compound assessed and subsequently monitoring viability 24 h posttreatment. A dose not eliciting substantial insect mortality (≤20%) after that period was selected for the treatments presented in this work.

### RNA Isolation

Isolation of small and large RNA fractions from insects exposed to hot and cold temperatures, or to insecticides of interest, was carried out using the mirVana miRNA Isolation kit (Thermo Fisher Scientific) following manufacturer's protocol and as described before (Bastarache et al. 2020). Isolates were prepared using two whole insects per replicate as starting material. RNA concentration of the isolates was obtained using a NanoVue Plus Spectrophotometer (GE Healthcare Life Sciences, Mississauga, ON, Canada). RNA samples were stored at −80°C until use.

### Synthesis of cDNA

The synthesis of cDNA was performed by mixing a volume of large RNA isolates corresponding to 1 μg, 1 μl of oligo dT, 1 μl 10 mM dNTPs, and a volume of DEPC-treated water for a final volume of 12 µl. The mixture was then incubated at 65°C for 5 min, followed by subsequent addition of 4 μl 5X first Strand Buffer, 2 μl 0.1 M DTT, and 1.5 μl of DEPC-treated water. The mixture was next incubated at 37°C for 2 min to which a volume of 0.5 μl M-MLV RT enzyme was added. Solution was ultimately incubated at 37°C for 50 min followed by 70°C for 15 min. The cDNA samples were stored at 4°C.

### qRT-PCR Amplification of Transcripts of Interest

Primers for amplification of Argonaute-1 (Ago1; LOC111515958), Argonaute-2a (Ago2; LOC111513702), Argonaute-3 (Ago3; LOC111510264), Dicer-2a (Dcr2a; LOC111517750), Dicer-2b (Dcr2b; LOC111509308), Exportin-5 (Expo-5; LOC111501848), Siwi-1 (LOC111515421), and Siwi-2 (LOC111505514) in *L. decemlineata* were designed using the Primer3Plus tool. Amplified fragment size of PCR products was approximately 215 base pairs. Primers are listed in Table 1. Semi-quantitative PCR was conducted by combining 5 µl of cDNA (10^−1^), 1 µl 25 µM forward primer, 1 µl 25 µM reverse primer, 5.5 µl of DEPC-treated water, and 12.5 µl of 2X Taq FroggaMix (FroggaBio, Concord, ON, Canada). Amplification began with a denaturing step at 95°C for 5 min, followed by 35 cycles at 95°C for 15 s, at temperature gradient between 54 and 65°C for 60 s and 72°C for 45 s. Agarose gel electrophoresis and sequencing at the Université Laval platform (Quebec City, QC, Canada) was completed to confirm product identity. Optimal hybridization temperatures for each primer pair and efficiency calculations were performed by qRT-PCR. Efficiencies were evaluated via amplification of a given target in serial dilutions of synthesized cDNA followed by slope measurement of a plot depicting Cq of amplified target versus quantity as reviewed elsewhere (Bustin and Huggett 2017). Quantification of transcript levels for each target was subsequently performed, in technical triplicates, using this approach. Volumes of 2.5 µl of cDNA (10^−1^), 0.5 µl DEPC-treated water, 1 µl 5 µM forward primer, 1 µl 5 µM reverse primer, and 5 µl of iTaq Universal SYBR Green Supermix (Bio-Rad, Hercules, CA) were mixed. Amplification protocol consisted of a denaturing step at 95°C for 3 min followed by at least 39 cycles at 95°C for 15 s and at optimal annealing temperature for 30 s. Transcript levels of α-tubulin or RP-18 were assessed in parallel reactions and used as references. Ultimately, this qRT-PCR approach was undertaken to evaluate the expression status of Ago1, Ago2, Ago3, Dcr2a, Dcr2b, Expo-5, Siwi-1, and Siwi-2 in *L. decemlineata* exposed to 15°C, 5°C, −5°C, and recovered at 15°C for the cold exposure experiment, in *L. decemlineata* submitted to 40°C and compared with control insects kept at 25°C for the heat exposure experiment, as well as in *L. decemlineata* exposed to the four insecticides of interest.

**Table 1.** Primers used for quantification by qRT-PCR of relevant transcripts

Primer		Sequence	Efficiency	Temperature
Ago1	Fwd	5′-GGTCGACCTATCGGACTGAA-3′	87.0%	61.0°C
	Rev	5′-GTTGCCAATAGGCAGTGGAT-3′		
Ago2	Fwd	5′-ACGATACCCAGCTTTCGATG-3′	108.3%	59.2°C
	Rev	5′-TTCAAATTGTGCAACGGTGT-3′		
Ago3	Fwd	5′-AGAAAGTCAGAAGCGGCTCA-3′	96.5%	60.5°C
	Rev	5′-CGTCTCTCCCCGTGTAAAAA-3′		
Dcr2a	Fwd	5′-GACAGCATGAAGGGTCACGA-3′	97.7%	60.5°C
	Rev	5′-TGGATTCCCTGGTGTCGTTG-3′		
Dcr2b	Fwd	5′-CGCTCGTACATTCAGTCGAA-3′	109.5%	59.0°C
	Rev	5′-AGGATCGGATCTCTCCTGGT-3′		
Expo-5	Fwd	5′-TAAGTACCAGGTCTCGGGCA-3′	98.5%	60.5°C
	Rev	5′-CGCCTCCCTCAACATCTCTC-3′		
Siwi-1	Fwd	5′-ACAGGTGCTATGTCGGTTGC-3′	95.6%	53.5°C
	Rev	5′-ATCTTGTGGCGTTTCTGTCC-3′		
Siwi-2	Fwd	5′-CGAGCTAGAGGAAGGGCAAG-3′	106.5%	60.5°C
	Rev	5′-TGAGTTGGTGTAGGCTGCTG-3′		
α-tubulin	Fwd	5′-GAGTTCCAGACCAACTTGGT-3′	107.9%	52.6°C
	Rev	5′- GCCATGTACTTGCCGTGACG -3′		
RP-18	Fwd	5′-TAGAATCCTCAAAGCAGGTGGCGA-3′	110.1%	60.0°C
	Rev	5′-AGCTGGACCACCGTGTTTCACTGC-3′		

Fwd (Forward) and Rev (Reverse) primers.

### dsRNA Synthesis

Primers for the synthesis of Ago2-targeting dsRNA were conceived based on *L. decemlineata* Ago2 sequence. Synthesis of dsRNA was performed using the MEGAscript RNAi Kit (Thermo Fisher Scientific) and following manufacturer's instructions. Forward: 5′-TAA TAC GAC TCA CTA TAG GGA GAG TTG GCG CCG AGA TAA ATA A-3′ and reverse: 5′-TAA TAC GAC TCA CTA TAG GGA GAT GGA ATT GGC CTT CTG AAA C-3′ primers were conceived with T7 sequences. PCR amplification of the target fragments was performed at 95°C for 5 min, followed by 39 cycles of 95°C for 15 s, 60°C for 30 s, and 72°C for 45 s. Purification of PCR products was performed using the QIAquick PCR Purification kit (QIAGEN, Hilden, Germany), sequenced as described previously and used as template for dsRNA production. Treatment of synthesized dsRNA was performed using DNase and RNase. Purified dsRNA was quantified on a NanoVue Plus Spectrophotometer and stored at −20°C until injection.

### dsRNA Injection

Injection of dsRNA was performed using a 10 µl Hamilton Microliter Syringe (Hamilton, Reno, NV). Plasticine was used to immobilize insects on their back for injection. Following *L. decemlineata* immobilization, a volume of 5 µl of a 600 ng/µl dsRNA solution was injected in the abdomen as exemplified elsewhere (Clements et al. 2017). Insects serving as controls were injected with an equal volume of saline solution used to dilute dsRNAs. The insects were subsequently incubated (Thermo Fisher Scientific) at 25°C under a 16L:8D photoperiod.

### Ago2 Silencing Analysis

Transcript target knockdown confirmation following dsRNA injection was assessed using qRT-PCR. RNA isolates were prepared with one whole insect per replicate using Trizol (Thermo Fisher Scientific) as per manufacturer's protocol. RNA concentration measurement and cDNA synthesis were described as above. Ago2 transcript levels were quantified via qRT-PCR using the Ago2 primers presented in Table 1. Primers were in a region not overlapping the one targeted by the dsRNA and towards the 5′ end of the targeted region. Quantification was conducted using an initial cycle at 95°C for 3 min, followed by 39 cycles of 95°C for 15 s and at optimal annealing temperature for 30 s. Transcript levels of RP-18 were used as reference and amplified in parallel reactions. Differences in Ago2 expression levels in saline- and dsRNA-injected insects were assessed with an unpaired Student's *t*-test.

### Cold Exposure Response in dsRNA-Injected *L. decemlineata*

The impact of dsRNA-mediated Ago2 knockdown was evaluated in saline- and Ago2 dsRNA-injected *L. decemlineata*. Adult insects were obtained in May 2021 from the Fredericton Research and Development Centre and were acclimated as outlined above. Knockdown efficiency assessment was performed in insects stored in liquid nitrogen seven to ten days following dsRNA injection. On the 14th day following the injections, insects (*n* = 22) from each group were subjected to progressively declining temperature exposures starting at 15°C for 2 h, 5°C for 2 h, −5°C for 2 h, and finally 15°C for 2 h. Insect viability was monitored following exposure to cold temperatures. Viability was assessed by initially providing insect with pieces of potato leaves to evaluate its propensity to feed, followed by subsequent shaking of the dish housing the insect, and evaluation of its ability to right itself as reported elsewhere (Jiang et al. 2012). Dead insects develop a grey complexion over time and this characteristic was also verified throughout the time-course to confirm mortality.

### Heat Exposure Response in dsRNA-Injected *L. decemlineata*

Injection of saline or dsRNA targeting Ago2 was initiated in adult *L. decemlineata*. An October 2020 batch of insects was provided from the Fredericton Research and Development Centre and was acclimated as outlined above. Seven days following injection, four insects from each group were stored in liquid nitrogen to be used for target knockdown validation. On the 14th day following the injection, insects (*n* = 23 saline and *n* = 22 Ago2) from each group were exposed to progressively increasing temperatures starting at 30°C for 2 h, 35°C for 2 h, and finally 40°C for 2 h. A second exposure to these temperatures, using comparable time durations, was performed 14 d after the initial temperature treatment. Insect viability follow-up was performed as described for cold exposure.

### Quantification and Statistical Analysis

CFX Manager software was used to collect quantification cycles and to confirm the presence of single peaks in qRT-PCR runs via melt curve analysis. Relative normalized transcript expression and statistical analysis of data were performed using CFX Maestro software version 1.1 (Bio-Rad). Outliers were identified using box-and-whisker plot. Significant differences between transcript levels in control insects versus treated insects were assessed by performing an unpaired Student's *t*-test (heat, insecticides, and knockdown experiments) or a one-way analysis of variance (ANOVA) with Bonferroni correction (cold experiments). Comparison of differences observed in survival curves was performed via log-rank test using GraphPad Prism version 8.0.2.

## Results

### Expression of Small Noncoding RNA-Associated Transcripts in *L. decemlineata* Exposed to Cold

**Fig. 1.**
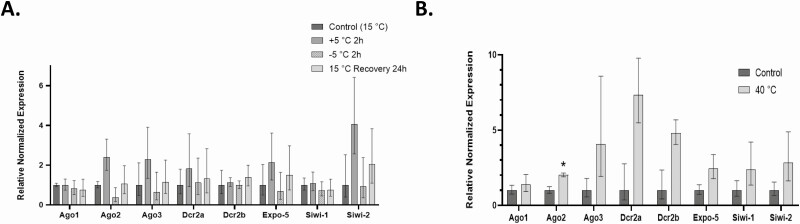
(A) Transcript levels in insects submitted to cold conditions versus control insects. Data are mean standardized transcript levels (mean ± SEM, *n* = 6 biological replicates). (B) Transcript levels in heat-exposed versus control insects. Data are mean standardized transcript levels (mean ± SEM, *n* = 3 biological replicates). Asterisks represent results significantly different from control samples (*P < 0.05).

Several targets of interest showed comparable trends and changes, albeit not significantly, in transcript levels expression at the four conditions investigated. Ago2, Ago3, and Siwi-2 were transcripts that displayed the most important increases with 2.40-, 2.28- and 4.05-fold changes in 5°C-exposed versus control insects, respectively. Transcript levels measured at −5°C proved to be more aligned with levels observed in control insects. Reduced transcript levels were nevertheless observed at that temperature with the most reduced levels observed for Ago2 and Ago3 that were 0.38-fold and 0.64-fold in −5°C-exposed insects when compared with levels detected in control insects, respectively. Expression status of these transcripts was stable in insects recovered at 15°C while transcript expression displayed increased Siwi-2 levels of 2.05-fold in these insects when compared with controls (Fig. 1A).

### Expression of Noncoding RNA-Associated Transcripts in *L. decemlineata* Exposed to Heat

Exposure to high temperatures elevated the expression status of Ago2 transcripts to levels in heat-exposed *L. decemlineata* that were 2.02-fold the ones observed in control insects (*P* < 0.05). Elevated levels were measured for most of the other transcript targets, including Ago3, Dcr2a, Dcr2b, Expo-5, Siwi-1, and Siwi-2, investigated in heat-exposed insects even though these changes were not significant. The strongest modulations were reported for Dcr2a and Dcr2b, which showed increases in transcript expression, albeit not significantly, of 7.32-fold and 4.80-fold in 40°C-exposed insects versus insects that were maintained at 25°C, respectively (Fig. 1B).

### Expression of Noncoding RNA-Associated Transcripts in *L. decemlineata* Exposed to Insecticides

**Fig. 2.**
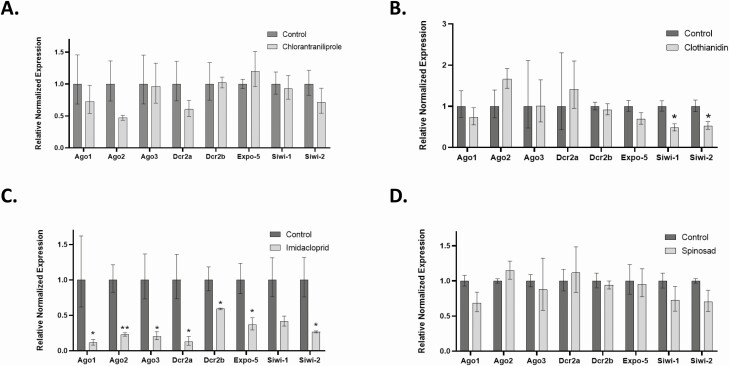
(A) Expression of transcript levels in chlorantraniliprole-treated versus untreated insects. Data are mean standardized transcript levels (mean ± SEM, *n* = 4–6 biological replicates). (B) Expression of transcript levels in clothianidin-treated versus untreated insects. Data are mean standardized transcript levels (mean ± SEM, *n* = 4–5 biological replicates). Asterisks represent results significantly different from control samples (*P < 0.05). (C) Expression of transcript levels in imidacloprid-treated versus untreated insects. Data are mean standardized transcript levels (mean ± SEM, *n* = 3 biological replicates). Asterisks depict results that are significantly different (*P < 0.05 and **P < 0.005). (D) Transcript levels in spinosad-exposed versus untreated insects. Data are mean standardized transcript levels (mean ± SEM, *n* = 4–5 biological replicates).

Characterization of expression status was performed in *L. decemlineata* exposed to chlorantraniliprole, clothianidin, imidacloprid, and spinosad. Transcript levels were quantified using qRT-PCR. Transcript levels were stable for most transcript targets assessed in chlorantraniliprole-exposed insects. Ago1, Ago2, and Dcr2a transcript expression was reduced, albeit not significantly, to levels that were respectively 0.73-fold, 0.47-fold, and 0.60-fold the values observed in control insects (Fig. 2A). Siwi-1 and Siwi-2 transcript levels were down-regulated in insects treated with clothianidin with levels of 0.48-fold (*P* < 0.05) and 0.52-fold (*P* < 0.05) the values observed in control insects (Fig. 2B). The rest of the investigated targets remained stable following treatment of *L. decemlineata* with clothianidin. On the other hand, most of the investigated transcripts displayed significant reduced expression levels in *L. decemlineata* exposed to imidacloprid. Measured expression of Ago1, Ago2, Ago3, Dcr2a, Dcr2b, Expo-5, and Siwi-2 revealed significant down-regulated transcript levels that were 0.11-fold (*P* < 0.05), 0.23-fold (*P* < 0.005), 0.20-fold (*P* < 0.05), 0.12-fold (*P* < 0.05), 0.59-fold (*P* < 0.05), 0.37-fold (*P* < 0.05), and 0.26-fold (*P* < 0.05) the values observed in control insects, respectively (Fig. 2C). Transcript levels exhibited stable expression in *L. decemlineata* exposed to spinosad for most of the targets measured. Ago1, Siwi-1, and Siwi-2 transcript expression was reduced, albeit not significantly, to levels that were respectively 0.69-fold, 0.72-fold, and 0.70-fold the values observed in control insects (Fig. 2D).

### Mortality of *L. decemlineata* in dsRNA-Injected Insects Exposed to Cold or Heat Challenges

**Fig. 3.**
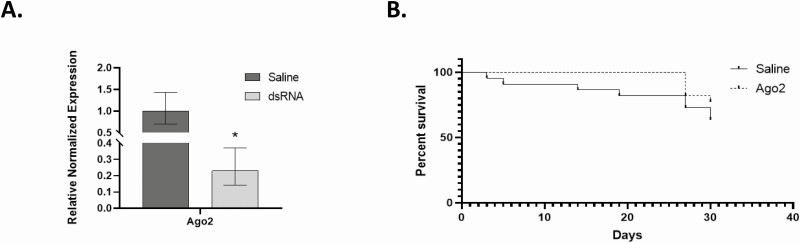
(A) Ago2 expression in *L. decemlineata* following dsRNA injection. Histogram shows Ago2 transcript levels in dsRNA- or saline-injected *L. decemlineata* postinjection. Data are mean standardized transcript levels (mean ± SEM, *n* = 7 biological replicates). Asterisks represent results significantly different from control samples (*P < 0.05). (B) Impact of cold response on insect viability in dsRNA-injected *L. decemlineata*. Kaplan–Meier survival analysis depicts *L. decemlineata* survival (*n* = 22) in saline- or Ago2 dsRNA-injected insects after exposure to temperatures ranging from 15°C, 5°C, −5°C and then returning to 15°C.

**Fig. 4.**
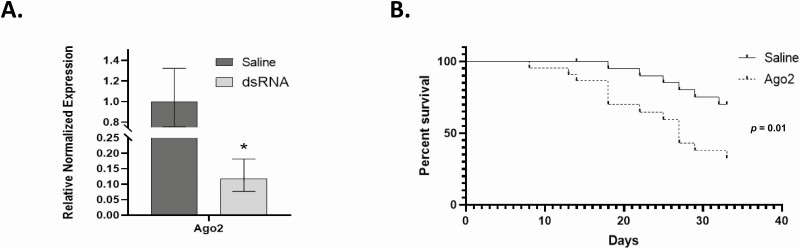
(A) Ago2 transcript levels in *L. decemlineata* following dsRNA injection. Histogram shows Ago2 expression status in dsRNA- or saline-injected insects postinjection. Data are mean standardized transcript levels (mean ± SEM, *n* = 4 biological replicates). Asterisks identify results significantly different from control samples (*P < 0.05). (B) Effect of heat exposure response on insect survival in dsRNA-injected *L. decemlineata*. Kaplan–Meier survival analysis presents *L. decemlineata* survival (*n* = 22–23) in saline- or Ago2 dsRNA-injected insects exposed to heat treatment (*P* = 0.01).

Ago2 was subsequently targeted considering the observed changes in its expression in *L. decemlineata* exposed to cold and hot treatments with the objective of assessing whether its modulation via dsRNA would impact the ability of insects to tolerate low or high temperatures. Cold treatment in insects with dsRNA-modulated Ago2 transcript levels was thus performed to assess the impact of Ago2 knockdown on *L. decemlineata* cold response. Reduced expression of Ago2 transcript levels was undertaken in *L. decemlineata* using dsRNA injection. Knockdown efficiency was subsequently evaluated using qRT-PCR. Ago2 transcript levels were significantly reduced in insects injected with dsRNA to levels that were 0.23-fold (*P* < 0.05) the ones observed in control insects (Fig. 3A). Cold exposure was subsequently performed in saline- and dsRNA-injected insects 14 d after the injection and insect survival was monitored. No significant variation was observed between the saline- and dsRNA-injected insects following exposure to cold temperatures (Log-rank test *P* = 0.26) (Fig. 3B). Heat exposure was also performed to assess the impact of Ago2 knockdown in saline- and dsRNA-injected *L. decemlineata*. Transcript levels of Ago2 were decreased significantly in dsRNA-injected insects to levels that were 0.12-fold (*P* < 0.05) the ones measured in insects injected with saline (Fig. 4A). On the 18th day following heat exposure, insect survival percentage was 95.0 % in saline-injected insects and 70.2 % in insects injected with Ago2-targeting dsRNA. Results collected at the end of the time-course, on the 33rd day following heat treatment, showed survival percentage of 70.0% in control insects compared to 32.4% in dsRNA-injected insects (Log-rank test *P* = 0.01) (Fig. 4B). In both cold and heat treatments, no mortality was detected in dsRNA-injected insects following dsRNA injection and prior to temperature exposures.

## Discussion

Multiple studies have reported the differential expression of small noncoding RNAs in insects exposed to varying temperatures. Examples include the identification of a signature of miRNAs that displayed changing levels in the overwintering *L. oryzophilus* (Yang et al. 2017) as well as profiling the differentially expressed miRNAs in larvae of the freeze-tolerant fly *Eurosta solidaginis* (Diptera: Tephritidae) (Lyons et al. 2016). Transcript levels of Ago1 and Ago2, key molecular players underlying miRNA and siRNA function, were also shown to be differentially expressed in response to select temperature treatments in the brown planthopper *Laodelphax striatellus* (Hemiptera: Delphacidae) (Zhou et al. 2016). While these reports support the modulation of the activity of small noncoding RNAs biogenic cascades, expression status of key players participating in these cascades is for the most part lacking. The current work showed increased transcript levels in *L. decemlineata* maintained at 5°C, including Ago2, during a cold exposure time-course. Adult *L. decemlineata* can cope with the cold temperatures associated with winter (Hiiesaar et al. 2001, Izzo et al. 2014) and the variation of such target in the current study is pointing towards a potential role for Ago2 before reaching sub-zero temperatures. It is noteworthy to add that others have explored the impact of varying cold acclimation durations, or incremental acclimation durations, on insect cold tolerance (Enriquez and Colinet 2019). It would be interesting to evaluate the influence, if any, of Ago2 in such an experimental setting in *L. decemlineata*. Ago2 transcript levels, as well as of Dcr2a and Dcr2b albeit not significantly, were also elevated in heat-exposed *L. decemlineata* in the current work. While previous work showed that levels of Dicer protein were increased following mild hyperthermia in select mammalian cell types (Oshlag et al. 2013), studies are sparse regarding modulation in insects of small noncoding RNA-associated targets in response to heat. Further exploration of the significance of Ago2 over-expression observed in *L. decemlineata* exposed to cold and hot temperatures was performed next. RNAi-based modulation of this target was conducted via dsRNA injection in adult beetles which were subsequently submitted to temperature treatments. It is interesting to note that similar approaches have been undertaken in insects to highlight the impact of transcripts of interest on cold response. Injection of dsRNA targeting myo-inositol-1-phosphate synthase (Inos) in *Drosophila montana* (Diptera: Drosophilidae) revealed a link between Inos and increased cold tolerance (Vigoder et al. 2016) while modulation of the capability (capa) neuropeptide gene via RNAi could impact chill coma recovery in *Drosophila melanogaster* (Diptera: Drosophilidae) (Terhzaz et al. 2015). RNAi-mediated knockdown of Ago2, a component of the RISC complex (Song and Joshua-Tor 2006), and Dcr2, a ribonuclease involved in siRNA synthesis (Elbashir et al. 2001), was successfully conducted in the western corn rootworm, *Diabrotica virgifera virgifera* (Coleoptera: Chrysomelidae) and mitigated the effects of lethal dsRNA consumption (Vélez et al. 2016). Protein levels of the former were increased following viral infection in *D. melanogaster* (Torri et al. 2020) while the latter has notably been associated with a role in stress defense in *D. melanogaster* (Lim et al. 2011) further supporting their likely relevance in stress mitigation. Even though RNAi-mediated Ago2 knockdown has been reported before in lepidopterans such as the European corn borer *Ostrinia nubilalis* (Lepidoptera: Crambidae) (Cooper et al. 2021), modulation of Ago2 to investigate its potential role in cold response has not been reported in *L. decemlineata* prior to this work. The current study showed, despite significant reduction in Ago2 transcript levels following dsRNA injection, no substantial change in *L. decemlineata* mortality rates in control versus injected insects following cold exposure. A similar approach revealed, on the other hand, a significant reduction in insect survival rates in Ago2 dsRNA-injected insects following heat exposure when compared with control insects exposed to a similar treatment. These results position Ago2 as a potential target of interest for heat response in *L. decemlineata*. Further investigation is thus envisioned to better delineate, if any, the function of Ago2 and other transcripts relevant to small RNA biogenesis for cold and heat adaptation in this potato pest. Given that Ago2 protein levels have been reported as modulated in conditions not evaluated here, such as infection procedure-related stress (Torri et al. 2020), status of targets of interest in additional stress should also be considered. Additional work to assess the optimal window to conduct cold and heat treatments in dsRNA-injected *L. decemlineata* would also be of interest.

Substantial changes were observed in *L. decemlineata* exposed to the neonicotinoid imidacloprid in this study. These results, including changes in Ago1 and Expo-5 transcript levels, underlie a potential variation in activity status of cascades leading to miRNA homeostasis as part of the molecular response to imidacloprid in this insect. Such changes in levels of noncoding RNAs following exposure to insecticides would align with previous work that demonstrated variations of 15 miRNAs in honeybee larvae sampled from imidacloprid-exposed hives when compared to control (Derecka et al. 2013). A study performed in adult honeybees exposed to the neonicotinoid dinotefuran for various time periods notably revealed a list of 27 differentially expressed miRNAs in insects treated with this compound for 10 d (Huang et al. 2021). Honeybees treated with the neonicotinoid thiamethoxam for the same duration displayed varying levels of seven miRNAs when compared with control insects (Shi et al. 2017). Honeybees exposed to the insecticide sulfoxaflor in combination with select pathogens displayed increases in Dicer-like gene expression (Al Naggar and Paxton 2021). Modulation of miRNAs in response to insecticides is also observed in *L. decemlineata*. Next-generation sequencing-based study revealed 14 upregulated and 19 downregulated miRNAs in this potato pest treated to imidacloprid (Morin et al. 2017b). Assessment of miRNA expression in different stages of *L. decemlineata* development revealed multiple conserved and novel miRNAs as well as identified putative roles underlying insecticide resistance for select miRNAs (Wiebe et al. 2020). While multiple reports support modulation of miRNAs in insects exposed to insecticides, information is sparse regarding differential expression of piRNAs following similar treatments. Pioneering work on the topic showed differential expression of piRNAs in mosquitoes *C. pipiens pallens* (L.) that were sensitive or resistant to the insecticide deltamethrin (Hong et al. 2014). Follow-up work highlighted the involvement of the piRNAs piRNA-3312 and piRNA-3878 as part of a pyrethroid response in this same species (Guo et al. 2017, Ye et al. 2017). RNAi-based approaches in *D. melanogaster* showed in female flies that modulation of Ago2 and Ago3, an important player in insect piRNA processing and function (Yamashiro and Siomi 2018), was associated with increased resistance to radiation exposure (Proshkina et al. 2021) supporting further investigation of piRNA expression and function in insect stress response. These studies warrant a closer investigation of the activity of small noncoding RNAs synthetic cascades with respect to insecticides response and resistance in *L. decemlineata*. In addition, fluctuating levels of the investigated transcripts measured in response to imidacloprid were not observed in insects exposed to the other insecticides studied supporting follow-up work to further explore the pattern of expression associated with these transcripts in *L. decemlineata* treated with different classes of insecticides.

The current study reported differential expression of multiple transcript targets associated with small noncoding RNA homeostasis in *L. decemlineata* exposed to diverse conditions. Several transcripts depicted variations notably resulting from treatments to the insecticides clothianidin and imidacloprid. Changes in Ago2 transcript levels were also observed in *L. decemlineata* exposed to heat and select cold temperatures. RNAi-mediated knockdown of Ago2 transcript levels was successful and was associated with variations of survival rates in control- versus dsRNA-injected insects exposed to heat. Subsequent work, to better understand the potential function of Ago2 in temperature response in this insect, is foreseen. RNAi-based modulation of additional targets of interest, in addition to Ago2, and that displayed varying levels in response to the challenges investigated in the current study should also be considered in parallel. Overall, these results provide novel information on the expression status of key players in small noncoding RNA synthesis and function as well as serve as a starting point for a broader characterization of these pathways in this insect.

## Supplementary Material

ieac004_suppl_Supplementary_Material
