# Supplementary figures and images for: Exposure to Temperature and Insecticides Modulates the Expression of Small Noncoding RNA-Associated Transcripts in the Colorado Potato Beetle, Leptinotarsa decemlineata (Coleoptera: Chrysomelidae)
Source: J Insect Sci. 2022 Feb 16;22(1):23. doi: 10.1093/jisesa/ieac004 (PMC8849280; doi:10.1093/jisesa/ieac004)

Figure S1.

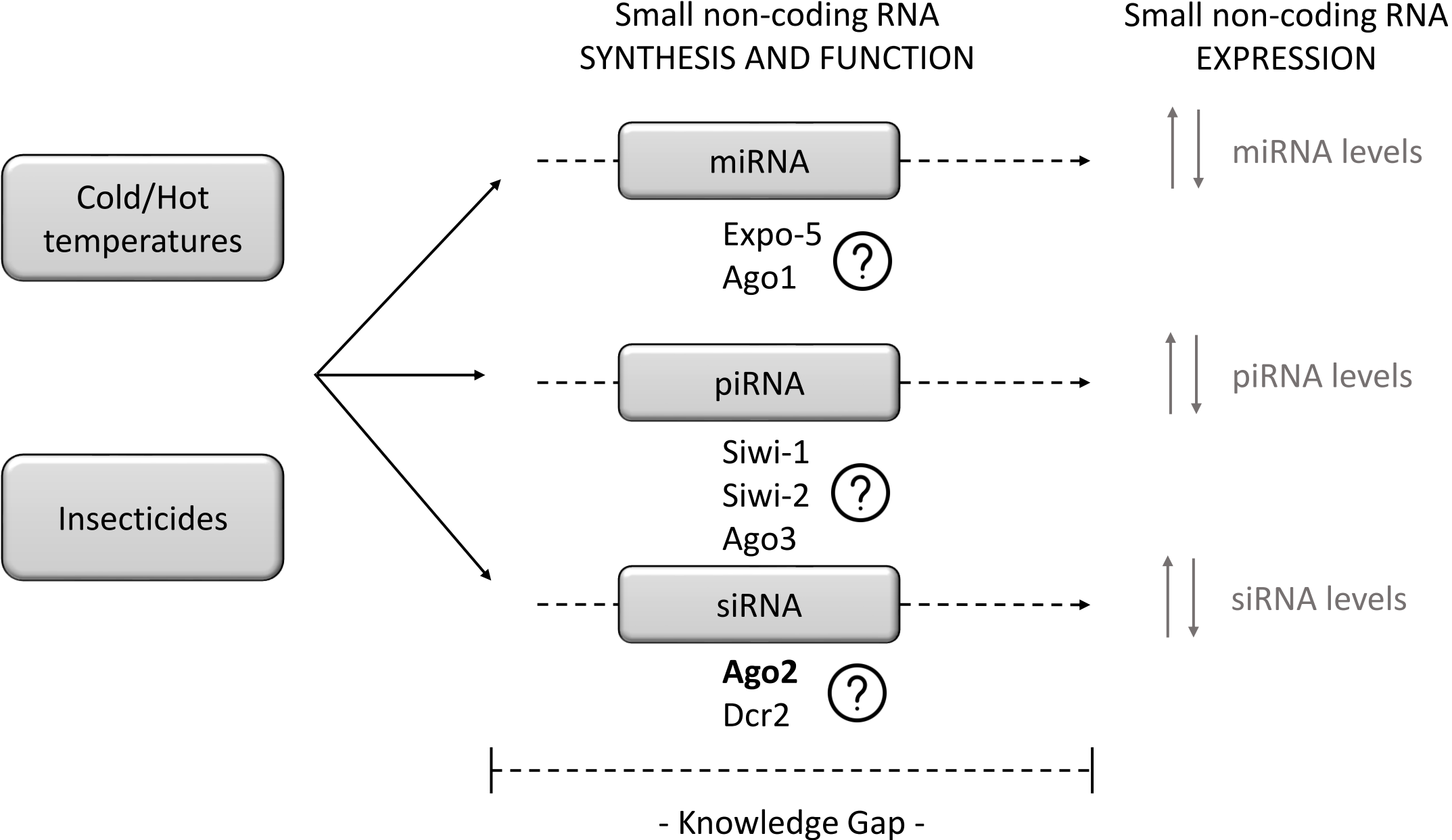

Supplement: ieac004_suppl_Supplementary_Material [file ieac004_suppl_supplementary_material.pdf]
